# Reply to “Comment on: A Systematic Review of Organic Versus Conventional Food Consumption: Is There a Measurable Benefit on Human Health? Nutrients 2020, 12, 7”

**DOI:** 10.3390/nu12030695

**Published:** 2020-03-08

**Authors:** Vanessa Vigar, Christopher Oliver, Carlo Leifert, Stephen P Myers

**Affiliations:** 1 NatMed Research, Southern Cross University, Lismore 2480, Australia; vanessa.vigar@scu.edu.au (V.V.); christopher.oliver@scu.edu.au (C.O.); 2 Integria Healthcare, Brisbane 4113, Australia; 3 School of Health and Human Sciences, Southern Cross University, Lismore 2480, Australia; 4 Centre for Organics Research, Southern Cross University, Lismore 2480, Australia; Carlo.Leifert@scu.edu.au; 5 Oliver Nutrition Pty Ltd., Lismore 2480, Australia

We would like to thank Di Renzo et al. for their interest in and comments on [1], our recent paper “A Systematic Review of Organic Versus Conventional Food Consumption: Is There a Measurable Benefit on Human Health?” [2].

Di Renzo et al. have commented that the “higher content of bioactive compounds and lower content of unhealthy substances” in organic foods contribute to maintaining an optimal health status and decreasing the risk of chronic degenerative non-communicable diseases. Whilst we, the authors, agree that this is likely to be the case (supported through observational research presented in our paper), the totality of the evidence supporting this assertion, we feel, is not yet definitive. However, in support of this argument, in addition to the positive evidence reported in several observational research studies, a discussion on the unknown safety of long-term pesticide consumption is given in the introduction of our review. Additionally, the “probably carcinogenic” classification by the World Health Organization of some pesticides and glyphosate [3] is highlighted in our discussion, suggesting that a reduction in these chemicals is highly likely to benefit health.

Secondly, the opinion of the authors concerning the randomisation that occurred in two papers cited by Di Renzo et al. [4,5] remains the same. As the trial treatments were not randomised, the improvement seen after the organic intervention may in fact have been caused by a four-week intervention of the Mediterranean diet over the course of the complete trial rather than just the final two-week intervention of the organic diet by itself.

Di Renzo et al. also raise concern that their research papers [4,5] were unfairly represented in our review as having “no organic certification defined”. They have rightly claimed that in Europe, organic production is regulated by law and therefore all products stated as organic must comply with this regulation. We acknowledge that this may have introduced a small negative bias against papers included in our review from European countries, which may have been reported as “no organic certification defined” where these reports did not specifically outline the labelling certification of the included dietary items. However, good scientific reporting should include information on organic certification in papers for international circulation. Some of the included papers in our review did report that organic products were appropriately certified [6,7,8,9]. We feel it is essential to clearly state that certified organic products were used for organic treatment groups in dietary intervention studies because there is concern (even in Europe and the USA) that some food products may be labelled as “*organic*” or “*from organic production*” (e.g., from farmers markets) although they are not from certified organic production [10,11].

After considering the comments made by Di Renzo and colleagues [1], we feel that our paper [2], as it stands, presents a balanced view of the current evidence on the potential health benefits of an organic diet.
